# Potential Role of Phosphoglycerol Dihydroceramide Produced by Periodontal Pathogen *Porphyromonas gingivalis* in the Pathogenesis of Alzheimer’s Disease

**DOI:** 10.3389/fimmu.2020.591571

**Published:** 2020-11-23

**Authors:** Chiaki Yamada, Juliet Akkaoui, Anny Ho, Carolina Duarte, Richard Deth, Toshihisa Kawai, Frank Nichols, Madepalli K. Lakshmana, Alexandru Movila

**Affiliations:** ^1^ College of Dental Medicine, Nova Southeastern University, Ft. Lauderdale, FL, United States; ^2^ College of Pharmacy, Nova Southeastern University, Ft. Lauderdale, FL, United States; ^3^ School of Dental Medicine, The University of Connecticut Health Center, Farmington, CT, United States; ^4^ Herbert Wertheim College of Medicine, Florida International University, Miami, FL, United States; ^5^ Institute for Neuro-Immune Medicine, Nova Southeastern University, Ft. Lauderdale, FL, United States

**Keywords:** Alzheimer's Disease, *Porphyromonas gingivalis*, dihydroceramides, amyloid precursor protein, tau protein, cellular senescence, senescence-associated secretory phenotype

## Abstract

**Background:**

Among different types of sphingolipids produced by human cells, the possible engagement of ceramide species in the pathogenesis of Alzheimer’s disease (AD) has attracted recent attention. While ceramides are primarily generated by *de novo* synthesis in mammalian cells, only a limited number of bacterial species, produce ceramides, including phosphoglycerol dihydroceramide (PGDHC) that is produced by the key periodontal pathogen *Porphyromonas gingivalis*. Emerging evidence indicates that virulence factors produced by *P. gingivalis*, such as lipopolysaccharide and gingipain, may be engaged in the initiation and/or progression of AD. However, the potential role of PGDHC in the pathogenesis of AD remains unknown. Therefore, the aim of this study was to evaluate the influence of PGDHC on hallmark findings in AD.

**Material and Methods:**

CHO-7WD10 and SH-SY-5Y cells were exposed to PGDHC and lipopolysaccharide (LPS) isolated from *P. gingivalis*. Soluble Aβ42 peptide, amyloid precursor protein (APP), phosphorylated tau and senescence-associated secretory phenotype (SASP) factors were quantified using ELISA and Western blot assays.

**Results:**

Our results indicate that *P. gingivalis (Pg)*-derived PGDHC, but not *Pg*-LPS, upregulated secretion of soluble Aβ42 peptide and expression of APP in CHO-7WD10 cells. Furthermore, hyperphosphorylation of tau protein was observed in SH-SY-5Y cells in response to PGDHC lipid. In contrast, *Pg*-LPS had little, or no significant effect on the tau phosphorylation induced in SH-SY-5Y cells. However, both PGDHC and *Pg*-LPS contributed to the senescence of SH-SY5Y cells as indicated by the production of senescence-associated secretory phenotype (SASP) markers, including beta-galactosidase, cathepsin B (CtsB), and pro-inflammatory cytokines TNF-α, and IL-6. Additionally, PGDHC diminished expression of the senescence-protection marker sirtuin-1 in SH-SY-5Y cells.

**Conclusions:**

Altogether, our results indicate that *P. gingivalis*-derived PGDHC ceramide promotes amyloidogenesis and hyperphosphorylation, as well as the production of SASP factors. Thus, PGDHC may represent a novel class of bacterial-derived virulence factors for AD associated with periodontitis.

## Introduction

Alzheimer’s disease (AD) is a multifactorial, highly heterogeneous, and complex neurodegenerative disorder that affects memory and cognitive functions leading to total dependence on nursing care at an advanced stage. Approximately 35.6 million patients are affected by AD worldwide and about 4.6 million new cases are added each year, causing enormous societal and economic burden (1, 2). It is commonly accepted that elevated amounts of aggregated Aβ peptides and hyperphosphorylated tau protein lead to deposition of extracellular amyloid plaques and intracellular neurofibrillary tangles in the brain of AD patients, making them hallmark features of AD neuropathology (3). The growing evidence suggests that age is the most prevalent risk factor for AD (4, 5). Although the age-associated gut bacterial dysbiosis is significantly correlated with the pathogenesis of AD (6), there is limited knowledge about the impact of oral bacteria on aging-associated AD.

The oral Gram-negative anaerobe, *Porphyromonas gingivalis*, is considered to be a keystone pathogen in chronic periodontitis (7–10). It is also well-documented that *P. gingivalis* is a contributory factor for various systemic diseases associated with aging, including type-II diabetes, and cardiovascular diseases (11). Furthermore, presence of *P. gingivalis* in AD brains (12, 13), as well as detection of elevated levels of IgG against *P. gingivalis* in periodontitis patients with AD, implicates a potential contributory role of this periodontal bacteria in the pathogenesis of AD (14).

*P. gingivalis* produces a wide variety of virulence factors of lipid origin, including lipopolysaccharide (LPS) and novel sphingolipids termed phosphoglycerol dihydroceramide (PGDHC) and phosphoethanolamine dihydroceramide (PEDHC) (15). Although ligation of *Pg*-LPS and PEDHC with Toll-Like Receptor (TLR) 2 and TLR4 elicits a strong inflammatory signaling induced in young mice, various published studies indicated that TLR function may be impaired in the context of aging (16–18). Furthermore, it was also recently demonstrated that *P.g*-LPS had little, or no, effect on the promotion of periodontitis inflammation induced in aged mice (19). We, however, reported that PGDHC ceramide promotes inflammation in a manner independent of TLRs (20), indicating that PGDHC may also represent a novel virulence risk factor that contributes to various age-related disorders, including periodontitis and AD.

Emerging evidence has indicated that among different sphingolipids, the levels of mammalian ceramide species were significantly elevated in brains of patients with more than one neuropathologic abnormality compared to the age-matched neurologically normal group of people (21). Although bacterial dihydroceramides, including those derived by *P. gingivalis*, share basic structural characteristics with mammalian ceramides, sphingolipid production by bacteria was thought to be a rare occurrence because only a limited number of gut and oral bacterial species can synthesize ceramides *de novo* (22–24). Nonetheless, because no studies have yet examined the role of ceramides produced by oral bacterial in the pathogenesis of AD, it remains unclear whether *P. gingivalis-*derived PGDHC contributes to the onset or progression of AD. Therefore, this study aimed to evaluate the potential involvement of PGDHC in the amyloidogenic processing of amyloid precursor protein (APP), hyperphosphorylation of tau, and cellular senescence, as key features of AD pathogenesis.

## Material and Methods

### Cell Cultures

Chinese hamster ovary-7WD10 (CHO-7WD10) cells stably expressing human wild-type amyloid precursor protein 751 (APP751WT) were cultured in DMEM media (Corning) supplemented with 10% fetal bovine serum (FBS), 1% penicillin/streptomycin, and 2mM L-glutamine. SH-SY5Y human neuroblastoma cells were cultured in 1:1 mixture of DMEM: F12 media with the same supplements as that of CHO-7WD10 cells.

### PGDHC and LPS From *Porphyromonas gingivalis*

PGDHC was isolated from *Porphyromonas gingivalis* (ATTC strain #33277) as previously described (20). For biological experiments, PGDHC was sonicated (2 min, 3 W) in phosphate-buffered saline (PBS) to achieve a concentration of 100 μg/ml. Ultrapure *Pg*-LPS was purchased from InvivoGen and prepared according to the manufacturer’s recommendation.

### Cytotoxicity Assays

CHO-7WD10 and SH-SY-5Y cells were seeded in a 96‐well plate at a density of 1 × 10^4^ cells/well and exposed to several concentrations of *Pg*-LPS and PGDHC (0, 1, 5, 8, 10 µg/ml). After 24 h of incubation, WST-1 metabolic activity assay (Sigma Aldrich) was employed according to the manufacturer’s instructions.

### Quantification of Soluble Aβ42

CHO-7WD10 cells were seeded in a 6‐well plate at a density of 1 × 10^6^ cells/well and cultured in the presence or absence of *Pg*-LPS or PGDHC for 48 h. Culture supernatants were collected and the amount of soluble Aβ42 was quantified using a commercial sandwich ELISA kit from Thermofisher.

### Quantification of β-Galactosidase Activity in SH-SY-5Y Cells

β-galactosidase activity was evaluated using a commercial senescence β-galactosidase staining kit (Cell Signalling Technology) according to the manufacturer’s recommendation. Staining-patterns of cells in the culture well were acquired by a 20x objective lens using an EVOS cell imaging system under bright-ﬁeld illumination.

### Western Blot Analysis

CHO-7WD10 and SH-SY-5Y cells were seeded at 1 × 10^6^ cells/well in a six-well plates and stimulated with various concentrations of PGDHC and Pg-LPS as listed for the cytotoxicity assay. After 48 h of stimulation, cells were lysed in the lysis buffer (Thermofisher) and protein concentration was measured using the BCA kit (Pierce). Next, proteins were separated using SDS/PAGE (Bolt 12% gel) electrophoresis, transferred onto a nitrocellulose (NC) membrane and blocked using iBlot2 (Thermofisher). The anti-mouse CT15 polyclonal antibody (1:500) (Calbiochem) was used for detection of full-length APP in CHO-7WD10.

To detect phosphorylated-Tau (p-Tau), sirtuin-1, and cathepsin B in SH-SY5Y cells, rabbit anti-p-Tau (Ser^396^), and -mouse AT1000 (Thr^212^/Ser^214^), - Sirt-1 and -cathepsin B polyclonal antibodies (1:1,000; Thermofisher) were used, respectively. The anti-human β-actin antibody (cat # 1:2,000; CST) was used to detect the levels of β-actin as a loading control. Finally, the membranes were washed with tris‐buffered saline (TBS) containing 0.05% Tween 20 and then processed using horseradish peroxidase (HRP)‐conjugated anti‐rabbit or anti‐mouse secondary antibodies (Amersham Pharmacia Biotech) followed by enhanced chemiluminescence detection (ThermoFisher). The signal intensity of Western blots was quantified using Image J.

### Real-Time PCR Analysis of Gene Expression

RNA was isolated using the PureLink™ RNA Mini Kit (Ambion, Life Technologies, USA) according to manufacturer’s instructions. Altogether, 1 μg of RNA was reverse transcribed with the Verso cDNA Synthesis Kit (Thermo Scientific). Gene expression was quantified using PowerUp™ Sybr™ Green Master Mix (Applied Biosystems Diagnostics) in the AriaMx Real-time PCR System (Agilent). Data were analyzed by the ΔΔCt method normalized to β-actin as the internal reference gene. Primer sequences are available upon request.

### Statistical Analysis

Significant differences in quantitative data were determined by one-way analysis of variance (ANOVA) followed by Tukey’s posthoc test using the paleontological statistics software (PAST) version 4.02 and *p* values ≤ 0.05 were considered significant. The data are displayed as means ± standard deviation (SD).

## Results

### PGDHC Enhances Secretion of Amyloid-Beta (Aβ) in CHO-7WD10 Cells

Since genetic, biochemical, and pathological evidence has strongly implicated that Aβ plays an early and crucial role in AD pathogenesis (25), we first tested whether PGDHC in comparison to *Pg*-LPS, exacerbate amyloidogenic processing of APP, using CHO cells stably expressing human APP751WT protein (CHO-7WD10) *in vitro*. When the concentrations of both *Pg*-LPS and PGDHC were greater than 5 µg/ml, our results showed that the viability of CHO-7WD10 cells was significantly reduced. Thus, CHO-7WD10 cells exposed to 8 and 10 µg/ml of PGDHC and *Pg*-LPS were excluded from the further examinations.

**Figure 1:**
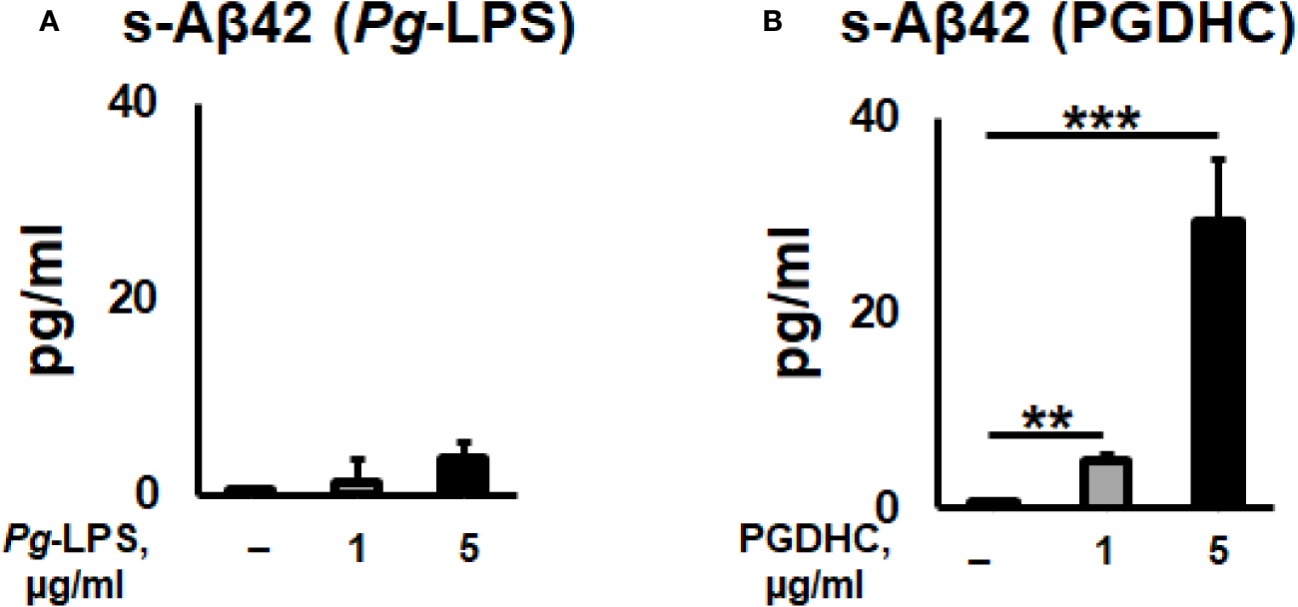
*Porphyromonas gingivalis*-derived phosphoglycerol dihydroceramide (PGDHC) promotes Aβ-42 secretion from Chinese hamster ovary (CHO) cells stably expressing human wild-type amyloid precursor protein 751 protein (CHO-7WD10). CHO-7WD10 cells were exposed to different concentrations of *Pg*-LPS **(A)** or PGDHC **(B)** for 48 h. Then, the conditioned media were collected and analyzed by ELISA. N = 4 samples/condition. **p < 0.01, ***p < 0.001.

**Figure 2:**
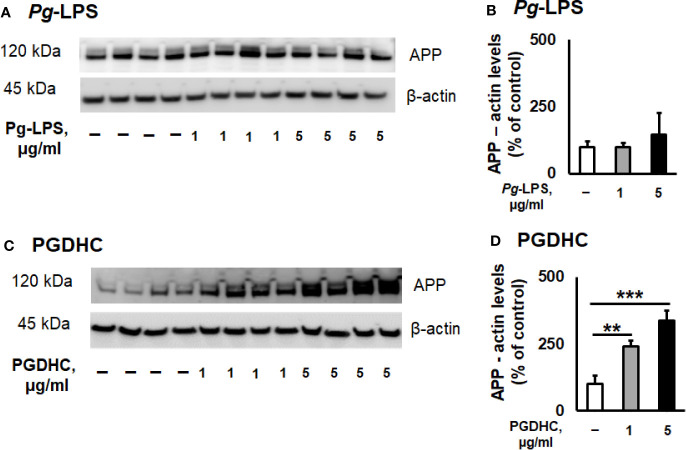
Phosphoglycerol dihydroceramide (PGDHC) amplifies the level of amyloid precursor protein (APP) in lysates of CHO-7WD10 cells. CHO-7WD10 cells were stimulated with various concentrations of *Pg*-LPS **(A, B)** or PGDHC **(C, D)** for 48 h and lysates were then prepared and analyzed by Western blot. The signal quantification was carried out using Image J. ANOVA with Tukey’s post-hoc test was used to evaluate the statistical significance. N = 4 samples/condition. **p < 0.01, ***p < 0.001.

According to our results, exposure of CHO-7WD10 cells to PGDHC, but not to *Pg*-LPS, significantly elevated the release of Aβ42 peptide in a dose-dependent manner (**Figures 1A, B**). Next, to detect the level of APP using a Western blot assay, lysates were prepared from the same cells that were used for Aβ42 quantitation after treatment with different concentrations of *Pg*-LPS and PGDHC. *Pg*-LPS had no or little effects on the APP levels in CHO-7WD10 cells (**Figures 2A, B**). By contrast, PGDHC significantly elevated the levels of APP in CHO-7WD10 cells when compared to the control, non-treated cells (**Figures 2C, D**). These observations suggested that PGDHC, but not *Pg*-LPS, enhances secretion of Aβ from CHO-7WD10 cells *in vitro*.

### PGDHC Induces the Site-Specific Phosphorylation of Tau (p-Tau) in SH-SY-5Y Cells

**Figure 3:**
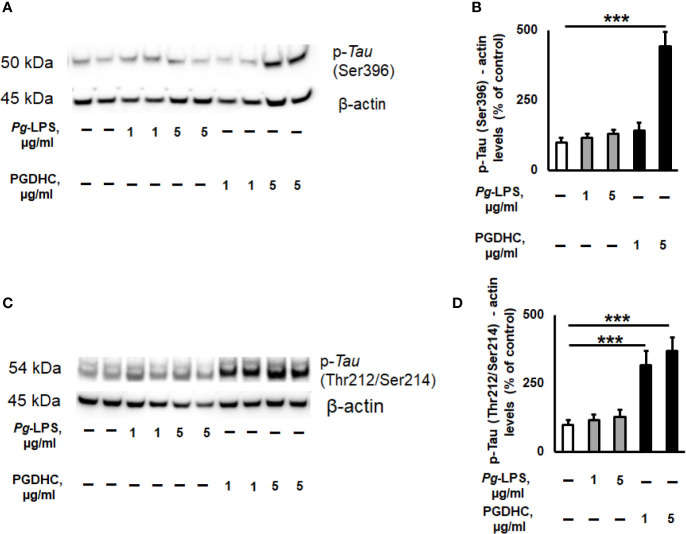
The effects of phosphoglycerol dihydroceramide (PGDHC) and lipopolysaccharide (LPS) isolated from *Porphyromonas gingivalis* on hyperphosphorylation of tau protein in SH-SY-5Y cells *in vitro*. Representative images and quantification of tau phosphorylation at Ser396 **(A, B)** and Thr212/Ser214 **(C, D)** loci in SH-SY-5Y cells after exposure to *P. gingivalis*-LPS (Pg-LPS) and PGDHC for 48 h. Cell lysates were prepared and analyzed by Western blot. Western blot signal quantification was done using Image J. ANOVA with Tukey’s post-hoc test was used to evaluate the statistical significance. n=4 samples/condition. ***p < 0.001.

Because published evidence demonstrated that the hyperphosphorylation of Tau protein was significantly upregulated in the hippocampi of AD patients (26), we next wanted to assess the p-Tau status in SH-SY-5Y cells exposed to various concentrations of *Pg*-LPS and PGDHC using two antibodies that recognize hyperphosphorylated tau at Ser^396^ and Thr^212^/Ser^214^ sites by Western blot assay. We observed that p-Tau at Ser^396^ was significantly increased after treatment with PGDHC when compared to control cells (**Figures 3A, B**). Furthermore, PGDHC also significantly upregulated p-Tau at Thr^212^/Ser^214^ in a dose-dependent manner compared to the control cells (**Figures 3C, D**). However, *Pg*-LPS did not induce p-Tau at either Ser^396^ or Thr^212^/Ser^214^ loci in SH-SY-5Y cells (**Figures 3A–D**). These results indicate that PGDHC may play an important role in the hyperphosphorylation of Tau protein, in addition to enhancing the secretion of Aβ.

### PGDHC Promotes the Development of Senescence-Associated Secretory Phenotype in SH-SY-5Y Cells

**Figure 4:**
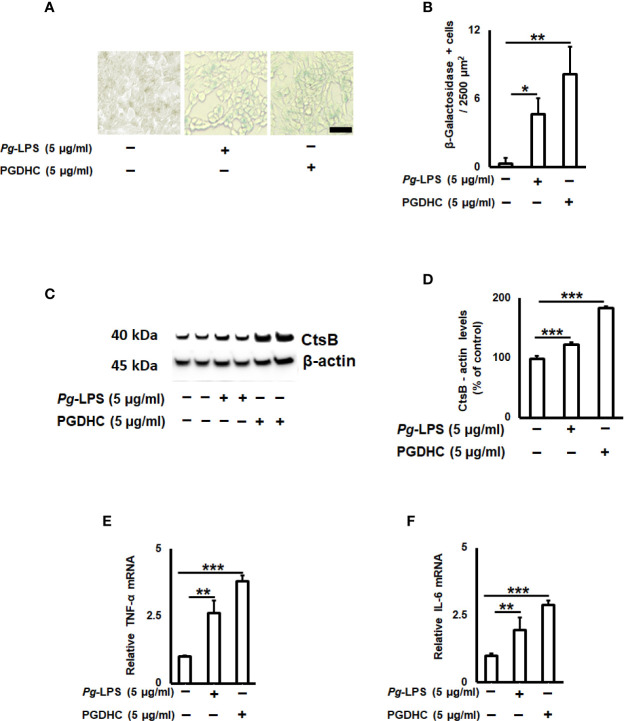
Quantification of senescence-associated secretory phenotype (SASP) factors on P. gingivalis-LPS (*Pg*-LPS) and phosphoglycerol dihydroceramide (PGDHC)-stimulated SH-SY-5Y cells *in vitro*. Representative images **(A)** and quantification **(B)** of β‐galactosidase activity. The number of blue β‐galactosidase positive senescent cells was quantified microscopically. Scale bar is 50 µm. Representative signals **(C)** and quantification **(D)** of cathepsin B. Expression patterns of TNF-a **(E)** and IL-6 **(F)** mRNAs in SH-SY-5Y cells exposed to *Pg*-LPS and PGDHC. ANOVA with Tukey’s post-hoc test was used to evaluate the statistical significance. N = 4 samples/condition. *p < 0.05, **p < 0.01, ***p < 0.001.

**Figure 5:**
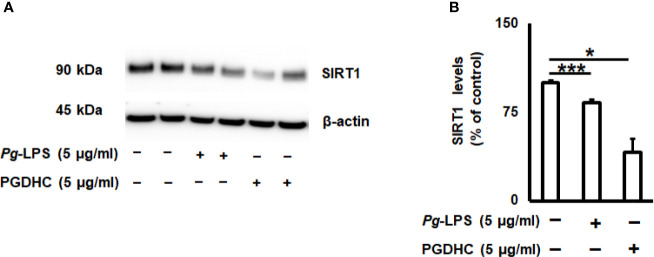
*Porphyromonas gingivalis*-derived phosphoglycerol dihydroceramide (PGDHC) abrogates expression of the senesce-protection sirtuin-1 (Sirt-1) in SH-SY-5Y cells *in vitro*. SH-SY-5Y cells were exposed either to Pg-LPS or PGDHC for 48 h and then the levels of Sirt-1 were evaluated by Western blot **(A, B)**. ANOVA with Tukey’s posthoc test was used to evaluate the statistical significance. N = 4 samples/condition. *p < 0.05, ***p < 0.001.

Since the cellular senescence of neurons is tightly connected with AD pathogenesis as well as other neurodegenerative diseases (27, 28), we next examined whether PGDHC or *Pg*-LPS elevated expression of some senescence-associated secretory phenotype (SASP) factors, including β-galactosidase, cathepsin B (CtsB) cysteine, and TNF-α and IL-6 pro-inflammatory cytokines in SH-SY5Y cells *in vitro*. We observed that exposure of SH-SY-5Y cells to *Pg*-LPS and PGDHC, both significantly elevated activity of senescence-associated β-galactosidase (**Figures 4A, B**) and CtsB (**Figures 4B, C**) compared with control cells. Further, expression patterns of pro-inflammatory TNF-α and IL-6 mRNAs were also significantly elevated in response to *Pg*-LPS and PGDHC (**Figures 4D, E**). On the other hand, expression of a senescence protection marker, sirtuin-1 (Sirt-1) was significantly diminished in SH-SY-5Y cells in response to *P. gingivalis*-derived PGDHC and *Pg*-LPS (**Figures 5A, B**). Therefore, these results indicate that persistent exposure of neurons to either PGDHC or *Pg*-LPS may induce phenotypes reminiscent of cellular senescence.

## Discussion

In this study, we aimed to examine the potential impact of phosphoglycerol dihydroceramide (PGDHC) isolated from the periodontal pathogen *Porphyromonas gingivalis* on the key features of AD pathogenesis, including amyloidogenesis, phosphorylation of tau protein, and cellular senescence, using *in vitro* models of AD. To the best of our knowledge, this is the first study reporting that PGDHC significantly upregulates amyloidogenesis in CHO-7WD10 cells. Our results also indicate that PGDHC elevates tau phosphorylation and expression of senescence-associated phenotype (SASP) factors in SH-SY-5Y neuronal cells *in vitro*.

Accumulating lines of evidence support the conclusion that ceramide sphingolipids are important structural and bioactive signaling molecules in mammalian cells, with significant roles in the regulation of cell apoptosis, senescence, and autophagy, leading to the development of AD pathogenesis as well as other age-related neurodegenerative disorders (29, 30). On the other hand, it was reported that a limited number of human gut and oral bacteria belonging to Bacteroidetes phylum are also able to produce dihydroceramides that upregulate intracellular host ceramide levels (23, 31). Further, bacterial-derived sphingolipids have been shown to signal *via* inflammation-related pathways in colon and gingival tissues (23, 24, 32). Important to this study, the gut Bacteroidetes species were detected at higher levels in AD patients compared to healthy controls (33). However, the role of dihydroceramides produced by oral *Bacteroides* spp. bacteria in the pathogenesis of AD has not been evaluated.

To date, unique dihydroceramides with non-mammalian structure, termed PGDHC and PEDHC, have been detected in three oral Gram-negative bacterial species associated with chronic periodontal disease, including *Porphyromonas gingivalis*, *Tannerella forsythia*, and *Prevotella intermedia* (24). These periodontal pathogens also produce several virulence factors, including LPS, gingipain, and lipids, which promote tissue inflammation, loss of connective tissue attachment, and bone loss (34, 35). It is important to mention that a recently published observation suggested that *P. gingivalis*-derived dihydroceramides are critical to the long-term persistence and presentation of other virulence factors, such as gingipains and polysaccharides (36).

Among periodontal bacteria species, *P. gingivalis* and its virulence factors were identified as significant risk factors for developing AD hallmarks (15, 37, 38). Increasing genetic, biochemical, and pathological evidence strongly implies that both amyloidogenesis and tauopathy play a crucial pathological role in brains of AD patients (25). It is commonly accepted that amyloidogenesis is associated with the production of Aβ peptides from its precursor protein APP by the consecutive actions of β- and γ-secretases, while tauopathy shows hyper-phosphorylation of tau protein in the brain of AD patients (39). A previous study reported that enhanced levels of intracellular mammalian ceramides directly affect the accumulation of *A*β peptides and p-Tau *in vitro* as well as *in vivo* (30). Here, we demonstrated that *P. gingivalis*-derived PGDHC increased the expression of APP protein and production of soluble *A*β42 peptide in CHO7W10 cells (**Figure 1**) as well as hyperphosphorylation of tau protein in SH-5Y-SY cells *in vitro* (**Figure 2**), indicating a potential contribution of oral bacterial-derived dihydroceramides in amyloidogenesis and tauopathy. By contrast, we observed no or minimal effects of ultrapure *Pg*-LPS on the release of soluble *Aβ*, and tau phosphorylation. These data contradict with earlier reports indicating that *Pg*-LPS promoted accumulation of Aβ and p-Tau *in vitro* as well as in the brains of young and mid-age APP-transgenic mice and their wild type (37, 40–42).

Besides the role of Aβ and p-Tau in the AD pathogenesis, a relationship between cellular senescence and AD may represent an additional hallmark in the context of aging (27, 43). More specifically, several groups have highlighted the potential beneficial effects of eliminating senescent cells on the AD-associated neurodegeneration (44–46). It was also demonstrated that elevated activities of lysosomal β-galactosidase and neurodegenerative cathepsin B (CtsB) as well as secretion of various pro-inflammatory cytokines, including tumor necrosis factor-α (TNF-α), interleukin-6 (IL-6), collectively termed as senescence-associated secretory phenotype (SASP) factors, directly correlate with cellular senescence and aging (47). Findings from our study also confirmed that both PGDHC and *Pg*-LPS exacerbate the activity of senescence-associated β-galactosidase as well as the levels of CtsB protein in SH*-*SY-5Y cells *in vitro* (**Figure 4**). In addition, we also demonstrated that PGDHC and *Pg*-LPS upregulated the expression of pro-inflammatory TNF-α and IL-6, suggesting the possible impact of *P. gingivalis*-derived virulence factors on the promotion of neuronal senescence (**Figure 4**). These data agree with earlier published observations indicating that LPS isolated from *P. gingvalis* induces premature cellular senescence (48) as well as promotes development of AD-like phenotypes in mice *via* a CtsB-dependent manner (42). Also, the pathological role of mammalian ceramides in the promotion of cellular senescence and aging has been well documented (49).

While earlier studies reported that elevated production of SASPs contributes to the AD pathology, it was suggested that the age-protection NAD^+^-dependent sirtuin enzymes display beneficial effects in aging-related disorders, including AD (50). A positive correlation between Sirt1 activity and reduction of Aβ plaques and tauopathies was established in various animal models of AD (51–54). Here, we also confirmed that PGDHC as well as *Pg*-LPS both diminished the amount of Sirt-1 protein in SH-SY-5Y cells (**Figure 5**), indicating that *P. gingivalis* may downregulate the expression of aging protection markers in the human brain. Since elevated expression of Sirt-1 reduces cellular senescence, the potential effect of sirtuin agonists to abrogate the negative effects of PGDHC in the pathology of AD warrants further examination.

## Conclusion

Collectively, the findings from this study indicate that PGDHC sphingolipid, isolated from the periodontal pathogen *P. gingivalis*, upregulated secretion of soluble Aβ42 peptide and expression of APP in CHO-7WD10 cells. Moreover, elevated hyperphosphorylation of tau protein (p-Tau) was observed in human neuronal SH-SY-5Y cells in response to PGDHC. Furthermore, we found that PGDHC contributed to the cellular senescence of SH-SY-5Y cells *via* 1) production of SASP markers, including beta-galactosidase, cathepsin B (CtsB), and pro-inflammatory cytokines TNF-α and IL-6, and 2) downregulation of the senescence-protection marker sirtuin-1 (Sirt-1). Altogether, these data indicate that PGDHC may be a novel class of bacterial-derived virulence factor for AD, finding which lay the groundwork for future studies, evaluating the molecular mechanisms of AD pathology associated with periodontitis.

## Data Availability Statement

The raw data supporting the conclusions of this article will be made available by the authors, without undue reservation.

## Author Contributions

AM and ML contributed to the conception, design of the study, and wrote the manuscript. CY, AH, JA, and CR performed all the experiments. RD, TK, and FN wrote sections of the manuscript. All authors contributed to the article and approved the submitted version.

## Funding

This work was supported by a Nova Southeastern University President Faculty Research Development Grant and NIH Grants R01AG-064003, K02AG-068595, R03DE-027153, R15DE-028699 (AM), R01DE-209709 (TK), and R01DE-027642 (FN).

## Conflict of Interest

The authors declare that the research was conducted in the absence of any commercial or financial relationships that could be construed as a potential conflict of interest.
