# Supplementary material for: Potential Role of Phosphoglycerol Dihydroceramide Produced by Periodontal Pathogen Porphyromonas gingivalis in the Pathogenesis of Alzheimer’s Disease
Source: Front Immunol. 2020 Nov 23;11:591571. doi: 10.3389/fimmu.2020.591571 (PMC7719741; doi:10.3389/fimmu.2020.591571)
Supplement: Supplementary file 1 [file DataSheet_1.docx]

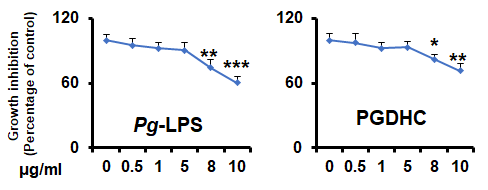


**Supplementary Figure 1.** The cytotoxicity of *Porphyromonas gingivalis*-derived LPS (*Pg*-LPS) and PGDHC on CHO-7WD10 cells *in vitro*. CHO-7WD10 cells were exposed to the different concentrations of *Pg*-LPS or PGDHC for 48 h and then were evaluated by WST-1 assay according to the manufacture's recommendation. N=4 samples/condition. **p*<0.05, ***p*<0.01, *** *p*<0.001.
